# Confined Chiral Microenvironments in Discrete Supramolecular Assemblies

**DOI:** 10.1021/cbe.6c00062

**Published:** 2026-07-20

**Authors:** Yingguo Li, Minghao Liu, Yan Liu, Chao Yu, Yong Cui

**Affiliations:** 1 School of Environmental and Chemical Engineering, 12676 Jiangsu University of Science and Technology, Zhenjiang, Jiangsu 212003, China; 2 School of Chemistry and Chemical Engineering, Frontiers Science Center for Transformative Molecules and State Key Laboratory of Metal Matrix Composites, 12474 Shanghai Jiao Tong University, Shanghai 200240, P. R. China

**Keywords:** chiral metallacycles, cages, self-assembly, coordination-driven, chiral transmission

## Abstract

Chirality is a fundamental feature of nature, governing the structure and function of biological systems. Translating molecular chirality into well-defined supramolecular architectures is crucial for understanding chiroptical phenomena and developing advanced chiral functional materials. In this Account, we summarize our research efforts over the past decade focused on the rational design and self-assembly of discrete chiral metallacycles and cages. We highlight how simple chiral building blocks can be programmed to spontaneously form complex two and three-dimensional architectures with confined chiral microenvironments. Our work begins with the precise construction of chiral metallacycles, including triangles, squares, and hexagons, where we demonstrate effective chirality transfer from the ligand to the metal center. These assemblies not only exhibit structural elegance but also serve as versatile platforms for studying chirality amplification. We then extend this strategy to three-dimensional chiral cages, including helicates, tetrahedra, octahedra, and nonclassical polyhedra. By engineering specific functional groups, such as NH, OH, or metal-salen sites within the confined cavities, we achieve precise control over the microenvironment. We further discuss the functional applications of these assemblies. The confined chiral spaces enable exceptional performance in enantioselective recognition, separation, and transmembrane transport, often mimicking the selectivity of natural enzymes. Moreover, we showcase how these cages function as supramolecular catalysts, where the confined nanospace significantly enhances reactivity and stereocontrol compared to nonencapsulated systems. Finally, we provide our perspective on the future challenges in this field, including the construction of nonclassical and defective structures, the hierarchical organization of discrete assemblies into extended materials, and the pursuit of biomimetic functions. This work aims to bridge the gap between molecular chirality and emergent supramolecular function, providing a comprehensive insight into the development of adaptive chiral materials.

## Graphical abstract

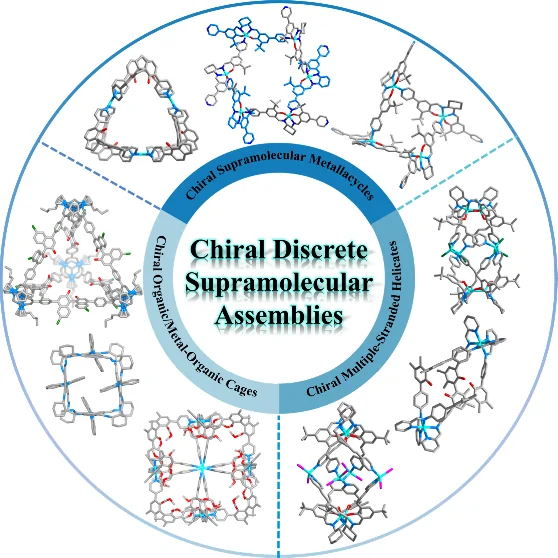

## 1 Introduction

Nature achieves remarkable specificity in biological processes through the confined chiral microenvironments of enzyme pockets, which precisely govern substrate binding and reaction selectivity.^1,2^ Inspired by these systems, supramolecular chemists have devoted extensive efforts to constructing artificial chiral assemblies, such as macrocycles^3–5^ and cages,^6–8^ capable of mimicking or even surpassing the functions of natural enzymes. These architectures have been widely applied in enantioselective recognition,^9–11^ separation^12,13^ and asymmetric catalysis.^14–17^

To emulate enzyme-like chiral cavities, a variety of chiral macromolecular systems have been developed.^18–22^ However, their practical advancement is often hindered by lengthy and synthetically demanding procedures. In this context, the utilization of chiral small molecules as modular building blocks for constructing supramolecular assemblies via multicomponent self-assembly offers a promising alternative.^23–26^ While individual small molecules typically lack well-defined three-dimensional nanospaces for substrate preorganization and transition-state stabilization, self-assembled macrocycles and cages provide precisely defined, tunable cavities and unique chiral microenvironments.^27–32^ These features endow distinct advantages in substrate encapsulation and transformation.

Over the past decade, our group has developed efficient strategies for the construction of chiral macrocycles and cages based on coordination-driven self-assembly and dynamic covalent chemistry.^33–37^ By rationally matching enantiopure ligands with appropriate metal ions or clusters, chiral information can be effectively transferred from molecular building blocks to well-defined supramolecular architectures. This strategy enables precise control over assembly topology, cavity size, internal functionality, and chiroptical properties.

Our systematic work has established a unified design paradigm for chiral metallacycles and cages centered on engineering confined microenvironments. Fundamental metallacycles enable efficient chirality transfer and amplification, where subtle conformational biases generate high efficiency enantioselective recognition and separation. Extending to three-dimensional chiral cages of helicates, tetrahedra, octahedra, and nonclassical polyhedra, we show that incorporating NH, OH, or metal-salen functionalities within confined cavities affords cooperative binding and exceptional enantiorecognition. These confined nanospaces also exhibit superior catalytic performance, leveraging intermetallic synergy and substrate preorganization to enhance reactivity and stereocontrol. Looking forward, we highlight challenges and opportunities in nonclassical and defective structures, hierarchical assembly into extended materials, and biomimetic functions. We believe readers will recognize that chiral supramolecular assemblies uniquely bridge molecular chirality with emergent functions, advancing adaptive chiral materials.

## 2 Structural Design

The design principles of coordination/dynamic covalent self-assembly provide a systematic framework for the construction of chiral macrocycles and cages. The core in this field is the “directional bonding” strategy, a concept pioneered and systematically developed by groups including Stang,^38,39^ Fujita,^40,41^ Nitschke,^42,43^ and Raymond,^44,45^ which has fundamentally shaped modern supramolecular design. We also leveraged this strategy to efficiently transfer chiral information from chiral centers to the supramolecular framework through the rational matching of organic ligands with appropriate coordination geometries and metal ions/monomer nodes, thereby enabling precise control over the assemblies topology, cavity microenvironment, and chiroptical properties. This review focuses on the key design strategies followed by our laboratory in the construction of chiral supramolecular assemblies. Specifically, we emphasize the synergistic modulation of self-assembly pathways and the chiral expression of final products through the chiral configuration of ligands, the spatial orientation of coordination sites, and the coordination modes of metal ions.

### 2.1 Chiral Metallacycles

Macrocycles, as a class of host with unique cavity structures, serve as important platforms for constructing host–guest systems in supramolecular chemistry. Inspired by the exceptional recognition properties of natural macrocycles, supramolecular chemists have devoted efforts to developing a wide variety of artificial macrocyclic hosts, including crown ethers, cyclodextrins, calix­[n]­arenes, cucurbit­[n]­urils, pillar­[n]­arenes, and other emerging macrocyclic frameworks.^38,46^ It is well-established that the host–guest recognition, sensing, and transmembrane transport functions of artificial macrocyclic systems are closely related to their cavity sizes, shapes, and internal microenvironments. Therefore, the rational design of macrocycles with tunable cavity sizes and specific functional groups should enable precise control over the selectivity of guest molecule recognition. However, in traditional stepwise synthetic approaches, this goal is often limited by lengthy synthetic routes and low yields. To overcome these limitations and achieve precise structural control, dynamic covalent chemistry or coordination-driven self-assembly strategies can be employed. With properly designed and preprogrammed building blocks, thermodynamically stable macrocyclic structures spontaneously assemble through reversible bond formation. This approach greatly simplifies synthetic procedures and provides new avenues for the rapid construction of structurally diverse and functionally tunable macrocycle libraries.

In this aspect, we have developed a series of chiral metallacycles, including three-membered,^47^ four-membered,^48,49^ six-membered^50–52^ and interlocked metallacycles.^24,25^ As illustrated in Figure 1a, two three-membered chiral metallacycles **1** and **2** (Pd_3_ **L** ^**1**^_6_ and Pd_3_ **L** ^**2**^_6_) were prepared in good yields by assembling of enantiopure ligand **L** ^**1**^ or **L** ^**2**^ (*R/S*-SPINOL) and Pd^2+^ in CH_3_CN. Single crystal X-ray diffraction (SC-XRD) unambiguously revealed the formation of chiral trimetallic coordination metallacycles.^47^ Each SPINOL ligand possesses the same chirality and coordinates to two Pd atoms via its two pyridyl groups, with an average Pd–Pd separation of approximately 10.3 Å. The cavities of **1** and **2** are decorated with methoxymethyl (MOM) and −OH groups, respectively. Notably, the −OH groups in **2** can serve as potential active sites for asymmetric catalysis.

**Figure 1:**
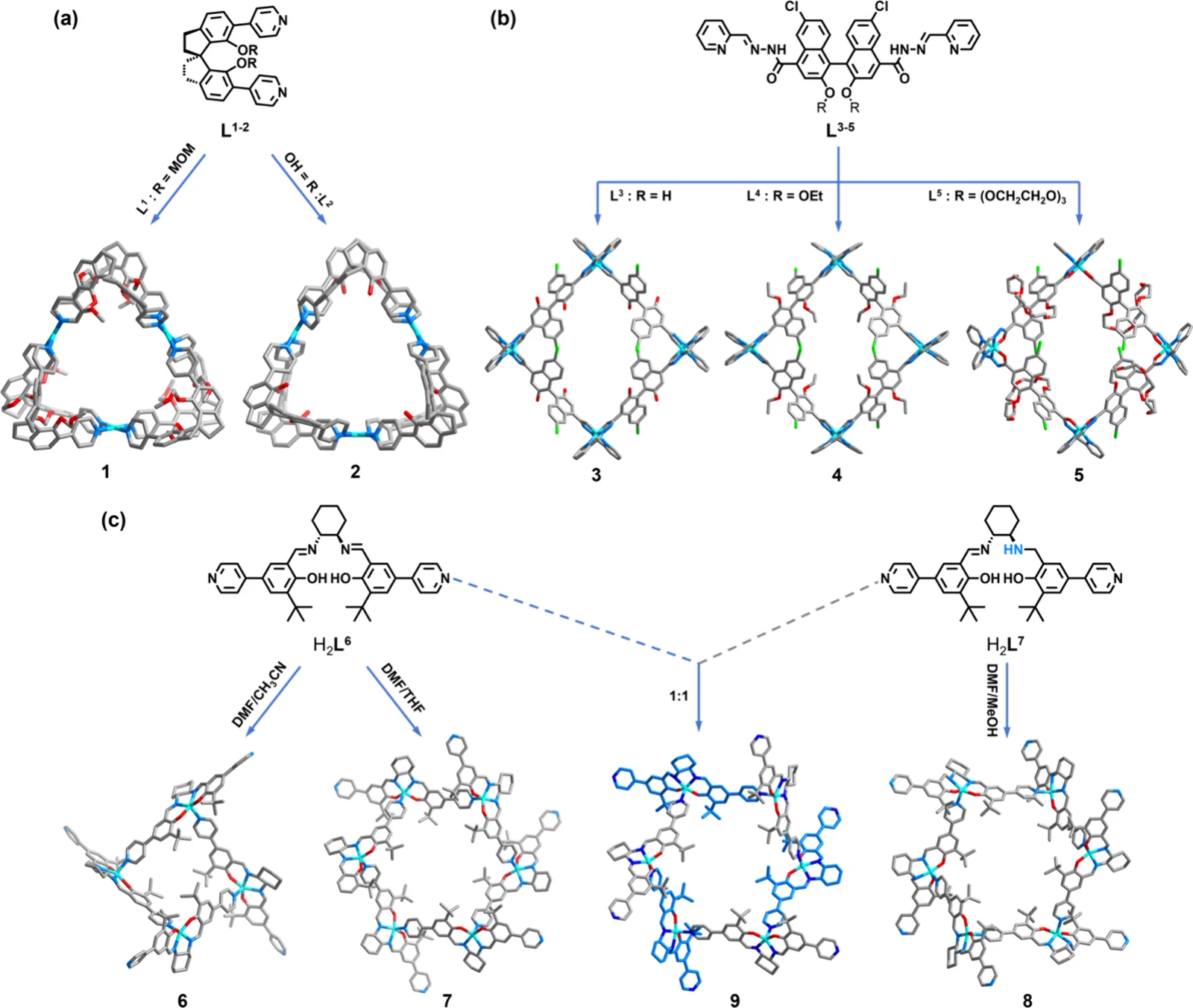
Structures of chiral metallacycles **1** and **2** (a), **3**–**5** (b), and **6**–**9** (c). Counterions and solvent molecules have been omitted for clarity; C: gray; N: blue; O: red; Cl: green; metal (Zn, Pd): cyan.

While the successful construction of trimeric metallacycles validated our design strategy for creating chiral microenvironments, we sought to expand the structural diversity and functional tunability of these systems. For example, engineering larger and more intricate assemblies offers extended cavities for the enantioselective recognition of larger chiral guests, which remains a challenge for smaller macrocyclic architectures. Motivated by the aforementioned work, we further employed the “directional bonding” assembly strategy to construct four-membered chiral metallacycles. Three four-membered metallacycles (**3–5**) with tunable pore microenvironments capable of selective anion transmembrane transport were successfully prepared by reacting chiral 1,10-bi-2-naphthol-based ligands (**L** ^**3**^-**L** ^**5**^) with Zn­(ClO_4_)_2_ with a molar ratio of 1:1 in CH_3_CN at room temperature (Figure 1b).^48^ In this system, the pore size and binding sites can be finely modulated by incorporating predesigned ligands bearing hydroxyl, ethoxyl, and pentaethylene glycol groups, thereby influencing the transmembrane transport properties of the metallacycles. The successful preparation of alkoxy-functionalized three- and four-membered metallacycles motivated us to explore other confined functional microenvironments. Furthermore, linearly chiral salen-type monomers proved suitable for constructing four-membered metallacycles. As depicted in Figure 1c, chiral metallacycle **6** was synthesized by the assembly of chiral ligand H_2_ **L** ^**6**^ and Zn^2+^ in DMF and CH_3_CN with the yield of 86%. The X-ray crystal structure revealed that **6** (Zn_4_ **L** ^6^_4_) is consist of four **L** ^**6**^ and four Zn^2+^.^49^ Four pairs of *tert*-butyl groups of the [Zn**L** ^6^] units are organized such that they partially cover two sides of the cyclic tetramer along its crystallographic 2-fold axis, generating four hydrophobic pockets, each occupied by one CH_3_CN.

Interesting, when the solvent of CH_3_CN was replaced with THF during the self-assembly process, the six-membered metallacycle **7** (Zn_6_ **L** ^**6**^_6_) was obtained.^50^ This solvent-induced structural transformation likely arises from thermodynamically controlled, self-correcting processes, suggesting that entropy plays a significant role in the formation of metallacycle. The X-ray crystal structure demonstrated that **7** is assembled from six [Zn**L** ^**6**^] units (Figure 1c). Each Zn center adopts a square pyramidal geometry, with the equatorial plane occupied by the N_2_O_2_ donors of one **L** ^**6**^ ligand and the apical position coordinated by a pyridine group from another **L** ^**6**^ ligand. Thus, each [Zn**L** ^**6**^] unit uses one terminal pyridyl group to bind a Zn atom, forming a hexamer, while the remaining pyridine group stays uncoordinated. Compared with the four-membered Zn_4_ **L** ^**6**^_4_, Zn_6_ **L** ^**6**^_6_ possesses a larger outer diameter of 4.01 nm and an inner pore diameter of 1.20 nm, enabling it to accommodate larger guest molecules. Beyond its discrete cavity features, the intermolecular packing reveals an even more intriguing supramolecular architecture. It should be noted that strong CH^**···**^π interactions between the butyl group and the conjugated pyridine ring of adjacent metallacycles direct the packing of metallacycles in parallel along the crystallographic *c*-axis, forming a nanoscale tubule with an interrupted channel. This implies that **7** can function not only as a discrete metallacycle for homogeneous molecular recognition and catalysis but also as a supramolecular nanotube for heterogeneous separation and catalysis.

To further exploit the potential of these metallacycles in enantioselective recognition, we try to introduce specific chiral binding sites into the framework. With this aspect, we designed a half-reduction salalen-type ligand (H_2_ **L** ^**7**^) with a chiral NH functionalities in the skeleton, which has a potential application in biological applications. Figure 1c shows that a similar approach can be applied to form metallacycle **8** (Zn_6_ **L** ^**7**^_6_) through the assembly of enantiomerically pure H_2_ **L** ^**7**^ with Zn^2+^. In this case, the exclusive formation of a hexameric complex across various solvent systems stands in sharp contrast to the self-assembly behavior of [Zn**L** ^**6**^]. X-ray crystallographic analysis revealed that **8** is isostructural with **7**, with bond lengths characteristic of salan (C-NH, 1.42–1.52 Å) and salen (C = N, 1.23–1.29 Å) motifs.^51^ The six inherently chiral NH sites are oriented toward the exterior of the cavity and are exposed to interstitial pores accessible to guest molecules. This structural modification was aimed to provide complementary interactions with both polar and nonpolar moieties, thereby affording synergistic hydrogen-bonding and hydrophobic effects for enantioselective recognition.

Given the structural similarity between H_2_ **L** ^**6**^ and H_2_ **L** ^**7**^, we further attempt to employ the mixture of H_2_ **L** ^**6**^ and H_2_ **L** ^**7**^ in different ratios to construct hybrid metallacyles. For example, using a 1:1 molar ratio of H_2_ **L** ^**6**^ and H_2_ **L** ^**7**^ led to the formation of the isostructural metallacycle **9** (Zn_6_ **L** ^**6**^_3_ **L** ^**7**^_3_) (Figure 1c). The crystal structure clearly reveals an alternating arrangement of three Zn**L** ^6^ and three Zn**L** ^7^ units, indicating the presence of three chiral NH binding sites within the structure. Moreover, by virtue of strong CH···π interactions between the butyl group and the conjugated phenyl ring of adjacent metallacycles, as well as intense π···π interactions between two face-to-face stacking rings from different metallacycles, the discrete species **6**-**9** can self-assemble into a robust, extended two-dimensional crystalline material. This 2D crystalline material can be exfoliated by sonication to yield free-standing monolayers approximately 2.3 nm thick.^52^ The crystalline chiral structure and surface morphology of this 2D material were unequivocally characterized by atomic force microscopy and high-resolution transmission electron microscopy. This progress underscores the potential applications of these fascinating metallacycles in both homogeneous and heterogeneous systems.

### 2.2 Chiral Cages

Chiral cages, characterized by their three-dimensional, well-defined cavities, offer a unique platform for molecular encapsulation and recognition. Unlike traditional macrocycles, these cage architectures provide a confined, isolated nanospace that can generate a distinctive chiral microenvironment and impose geometric restrictions, which are fundamentally different from those in bulk solution. Such confined spaces can alter the physical and chemical properties of encapsulated guests, including their reactivity and spectroscopic behavior. Notably, chiral cages constructed from dynamic covalent bonds or coordination-driven assembly have gained considerable attention due to their tunable cavities, inherent chirality, and structural robustness.^53–55^ Similar to chiral microenvironments found in enzyme active sites, the well-organized interior of these cages can facilitate enantioselective recognition, asymmetric catalysis, and selective separation processes. If the internal cavity of a chiral cage can be further engineered with specific functional groups or helical conformations, it could mimic the sophisticated functions of biological receptors, leading to significant advances in chiral materials and biomimetic chemistry.

#### 2.2.1 Chiral Helicates

In the initial stage of this study, chiral salan ligands were employed as subcomponents for the construction of helicate cages, owing to their structural flexibility and abundance of hydrogen-bonding sites. Heating the ligands of H_2_ **L** ^**8**^ or H_2_ **L** ^**9**^ with Zn^2+^ in a mixed solvent system afforded helicate cages **10** and **11** ([Zn_8_ **L**_4_]), respectively (Figure 2a). SC-XRD analysis revealed that cages **10** and **11** feature with a typical quadruple-stranded helicate structure, and the basic building block is a Zn_2_ **L**_2_ dimer built of Zn**L** monomer.^56,57^ Each dimetallic core consists of two five-coordinate, trigonal-bipyramidal Zn­(II) centers encapsulated within N_2_O_2_ pockets and bridged by two phenolate oxygen atoms. Consequently, the dimeric unit is stabilized by two equivalent coordination bonds, forming a four-membered Zn_2_O_2_ ring. A pair of bowl-shaped Zn_2_ **L**_2_ dimers are further interconnected via their peripheral pyridyl groups through coordination to four Zn centers located in the equatorial plane. Moreover, strong intermolecular π···π, CH···π and CH···X (X= Cl, I) interactions direct the packing of these helicates into porous three-dimensional architectures, analogous to the previously reported salan-based metallacycles.

**Figure 2:**
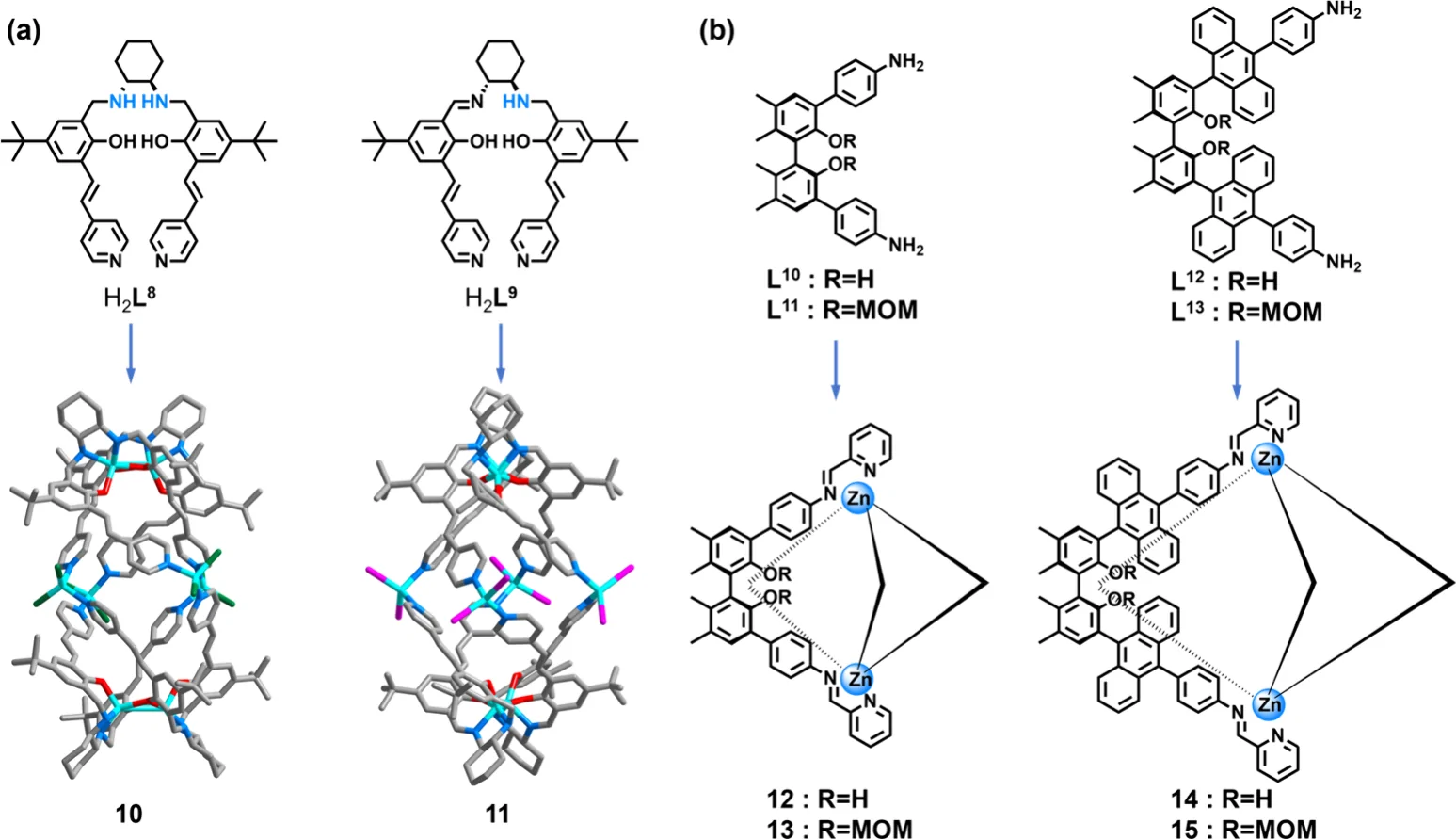
Structures of chiral helicates **10** and **11** (a). Self-assembly of triple-stranded helicates **12–15** (b). C: gray; N: blue; O: red; Cl: green; I: purple; Zn: cyan.

As an important branch, triple-stranded helicates have attracted considerable attention due to the ease with which their helical handedness, cavity size, and electrochemical properties can be modulated through the rational design of organic ligands and the choice of suitable metal ions. In this context, we developed a new system for the self-assembly of well-defined chiral triple-stranded helicates employing axially chiral 1,1′-biphenyl-type ligands (**L** ^**10**^-**L** ^**13**^). The reactions of ligands **L** ^**10**^-**L** ^**13**^ with 2-formylpyridine and Zn^2+^ in CH_3_CN at 70 °C produced helicates **12**-**15** with the general formula [(Zn_2_ **L**_3_)­A_4_] (where A = counteranion) as the uniquely observed products (Figure 2b). SC-XRD analysis of **12** confirmed the formation of a helical triple-stranded complex, which comprises three **L** ^**10**^ strands and two Zn^2+^ ions. The two Zn­(II) centers occupy the vertices, while three bis-bidentate ligands helically wrap around and coordinate to them.^58^ Each metal center adopts a pseudo-octahedral six-coordinate geometry with bidentate chelating moieties, exhibiting an average Zn–N bond length of 2.15 Å. Notably, cage **12** features a nanosized cavity measuring 12.5 Å × 4.8 Å × 4.8 Å and an entrance window of 6.3 Å × 5.4 Å, which accommodates guest molecules.

Remarkably, **10** and **12** displayed more than a 3-fold increase in optical rotation compared to their ligand precursors, implying that such helical-shaped supramolecular architectures can indeed generate unexpectedly strong chirality amplification. This property is particularly advantageous for recognizing guest molecules that differ only subtly in their structural features.

#### 2.2.2 Chiral Polyhedral Cages

In contrast to the structural flexibility of helicates, polyhedral cages always possess rigid cavities which are more amenable to rational design and predictable self-assembly, as their geometries can be preprogrammed through the careful selection of metalloligands and organic linkers with fixed coordination angles. Here, we will focus on the discussion of chiral tetrahedral, octahedral, and other types of polyhedral cages.

##### 2.2.2.1 Chiral Tetrahedral Cages

As shown in Figure 3, several chiral tetrahedral cages based on BINOL or biphenyl scaffolds were synthesized via self-assembly. Reaction of **L** ^**14**^ with M^III^ chloride (3:2 molar ratio) in DMF, followed by layering the solution with methanol, afforded the desired complexes M_4_ **L**_6_ (M = Fe for **16**, Ga for **17**) in moderate yields (Figure 3a). SC-XRD studies on **16** and **17** unambiguously revealed the formation of nanosized chiral cages with cavity volumes of approximately 1800 Å^3^.^59^ In each polyhedron, the metal centers adopt a nearly ideal octahedral geometry, coordinated by three chelated acac anions from three distinct ligands. Each of the six ligands possesses the same handedness of chirality and bridges two metal centers through its two chelating acac groups, with an average metal–metal separation of about 12.0 Å for both **16** and **17**. The pendant MOM groups are well arranged on the cage surface, promoting the formation of relatively enclosed chiral cavities. However, single metal nodes often suffer from limited structural stability. To address this issue, a *C*_3_-symmetric trinuclear zirconium cluster was introduced into the cage framework to enhance structural robustness. Linear bidentate BINOL-derived ligands (H_2_ **L** ^**15**^-H_2_ **L** ^**17**^)^60^ and a BINAP-derived ligand (H_2_ **L** ^**18**^)^61^ were prepared and reacted with bis­(*n*-butylcyclopentadienyl)­zirconium dichloride (*n*-Bu_2_-Cp_2_ZrCl_2_) to yield single crystals of cages **18**–**21** with the general formula [(nBu-Cp)_3_Zr_3_(μ_3_-O)­(μ_2_–OH)_3_]_4_(**L**)_6_ as the uniquely observed product (Figure 3b,c). SC-XRD analysis revealed that each cage consists of six linear **L** ligands connected by four (nBu-Cp)_3_Zr_3_(μ_3_-O)­(μ_2_–OH)_3_ vertices, forming a tetrahedral nanospace. Notably, 12 functional groups are oriented toward the interior of the cavity, thereby generating a superior chiral microenvironment for biomolecular recognition and binding.

**Figure 3:**
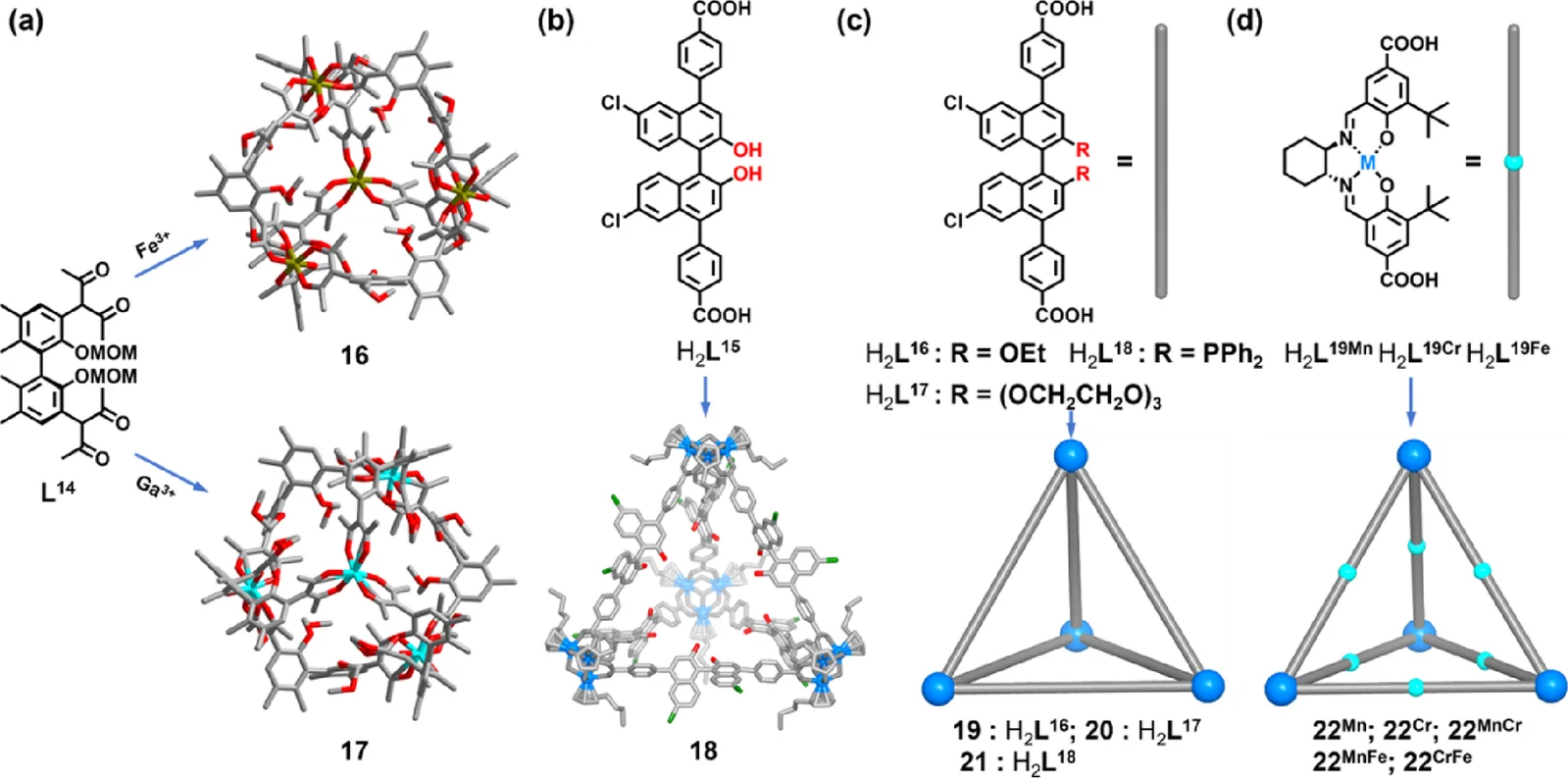
Self-assembly and crystal structures of chiral tetrahedral cages **16–17** (a) and **18** (b). Schematic representations of Binol-based tetrahedral cages **19**–**21** (c) and Salan-based tetrahedral cages **22** ^Mn^, **22** ^Cr^, **22** ^MnCr^, **22** ^MnFe^, and **22** ^CrFe^ (d). Counterions and solvent molecules have been omitted for clarity; C: gray; O: red; Cl: green; Fe: brown; Ga: cyan; Zr: blue.

Furthermore, metallosalen-based ligands (H_2_ **L** ^**19Mn**^, H_2_ **L** ^**19Cr**^, and H_2_ **L** ^**19Fe**^) were also employed to assemble tetrahedral cages with trinuclear zirconium clusters (Figure 3d). Heating a mixture of Cp_2_ZrCl_2_ and H_2_ **L** ^**19M**^ (M = Mn^3+^ or Cr^3+^) in DMF and THF at 65 °C afforded the cages in yields of approximately 70%. Their well-defined structures were unambiguously characterized by using a variety of techniques, including SC-XRD, ^1^H NMR spectroscopy, and ESI-MS. SC-XRD showed that cages **22** ^**Mn**^ and **22** ^**Cr**^ are constructed from four trinuclear zirconium clusters and six ligands.^62^ Each cage possesses 12 hydroxyl groups that engage in 12 O–H···Cl···H–O intercluster hydrogen-bonding interactions with adjacent clusters, thereby facilitating the formation of chiral three-dimensional porous structures. Interestingly, in this system, the catalytic metal species (M = Mn^3+^, Cr^3+^, and Fe^3+^) could be precisely tuned within the confined nanospace. For instance, cage **22** ^**MnFe**^ contains both Mn^III^(salen) and Fe^III^(salen) moieties, which can be readily prepared via a mixed-linker strategy. Such heterometallic cages provide enhanced complexity and synergism for sequential asymmetric reactions with remarkable catalytic performance.

##### 2.2.2.2 Chiral Octahedral Cages

Having established structurally robust tetrahedral platforms, we envisioned that a strategic expansion of the cavity dimensions would not only accommodate bulkier substrates but also facilitate more intricate catalytic transformations. Guided by this design principle, we constructed a series of octahedral cages tailored for asymmetric catalysis. In this context, a tetranuclear metal cluster was employed to assemble with bidentate or tridentate ligands to yield octahedral cages (Figure 4). For example, SC-XRD revealed that chiral octahedral cage **23** consists of six [Zn_4_(μ_4_-O)­(TBSC)] (TBSC = *p*-*tert*-butylsulfonylcalix­[4]­arene) clusters as vertices and 12 H_2_ **L** ^**19Mn**^ ligands as edges. The overall dimensions of the cage are approximately 43.4 × 44.0 × 44.3 Å^3^, and the inner cavity measures 28.4 × 28.4 × 28.4 Å^3^.^63^

**Figure 4:**
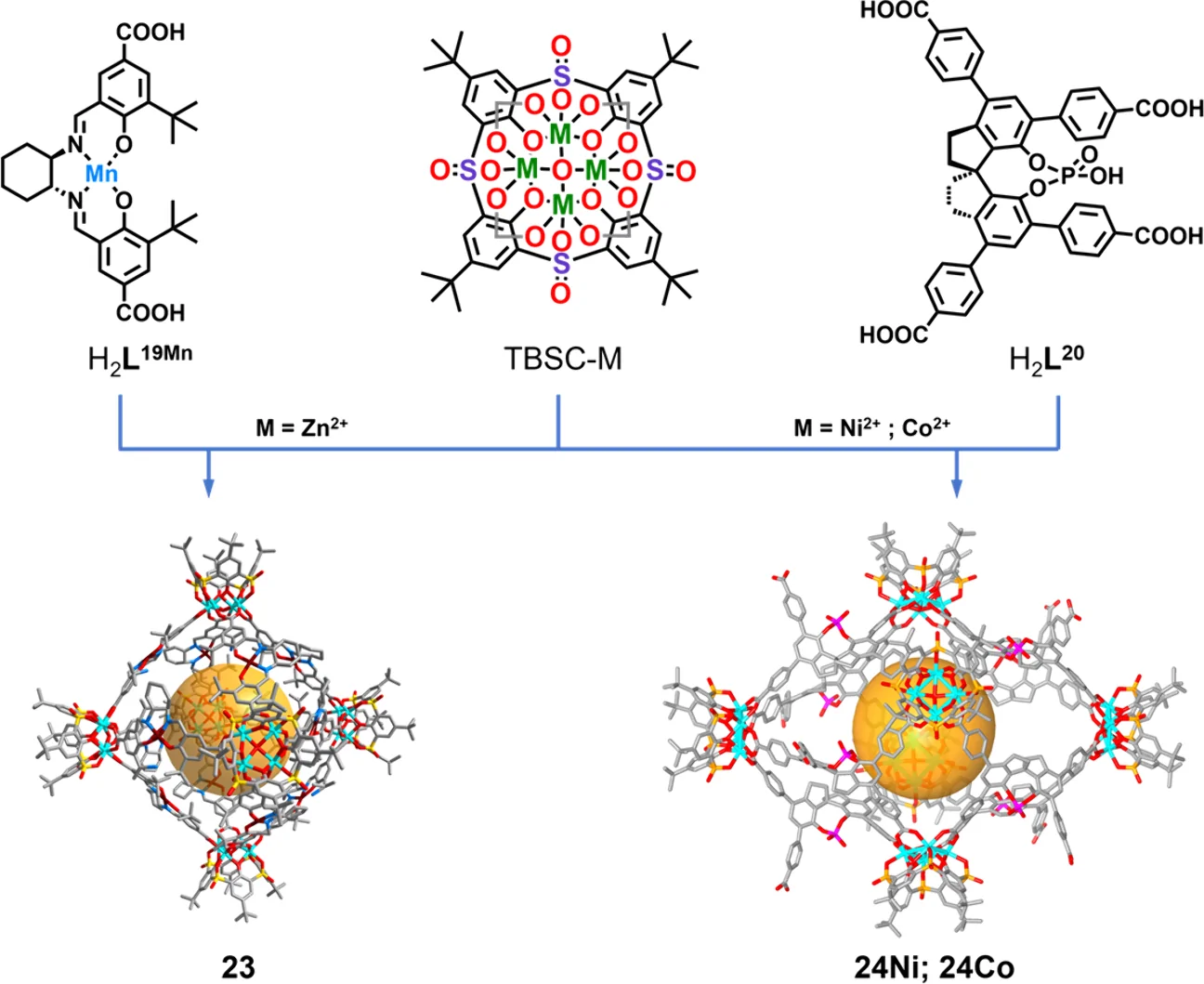
Self-assembly and crystal structures of chiral octahedral cages **23** and **24**. Counterions and solvent molecules have been omitted for clarity; C: gray; N: blue; O: red; S: yellow; P: purple; metal (Zn, Ni, Co): cyan.

Furthermore, the chiral SPIONL ligand (H_4_ **L** ^**20**^) was also applied to construct a chiral phosphoric acid–based cage. Heating a mixture of H_4_ **L** ^**20**^, H_4_TBSC, and NiCl_2_·6H_2_O or Co­(NO_3_)_2_·6H_2_O (1:1:10 molar ratio) in DMF and H_2_O afforded rectangular-shaped crystals of cages **24Ni** and **24Co** with the formula {[M_4_(μ_4_-H_2_O)­(TBSC)]_6_[HL]_8_}·G (M = Ni and Co for **24Ni** and **24Co**, respectively) in good yields. SC-XRD showed that cage **24Ni** is composed of six shuttlecock-like tetranuclear Ni_4_-TBSC nodes and eight H**L** ^**20**^ linkers.^64^ Notably, in the ligand H_4_ **L** ^**20**^, only three carboxyl groups participate in coordination to construct the cage, while the remaining carboxyl group points outward. Consequently, each octahedral cage features eight benzoic acid groups on its surface, rendering them amenable to postsynthetic modification.

##### 2.2.2.3 Chiral Cubic Cages

Beyond tetrahedral and octahedral geometries, we further extended our design strategy to cubic architectures, which offer distinct symmetry and host–guest properties. To construct cubic cages, flexible ligands **L** ^**21**^ and **L** ^**22**^ were selected to react with Pd^2+^ in CH_3_CN (Figure 5a). SC-XRD revealed that cage **25** exhibits noncrystallographic cubic symmetry and is constructed from six *C*_4_-symmetric palladium ions and 12 chiral banana-shaped biphenol-based linkers. All palladium ions are located at the center of each face of the cubic cage. The crystal structure of cage **26** was similar to that of **25**, with the MOM group replacing the crown ether group. Interestingly, the crown ether groups embedded in cage **26** can act as a capture platform to trap BODIPY dye in its potassium salt form, thereby facilitating the generation of circularly polarized luminescence in these host–guest complexes.^65^

**Figure 5:**
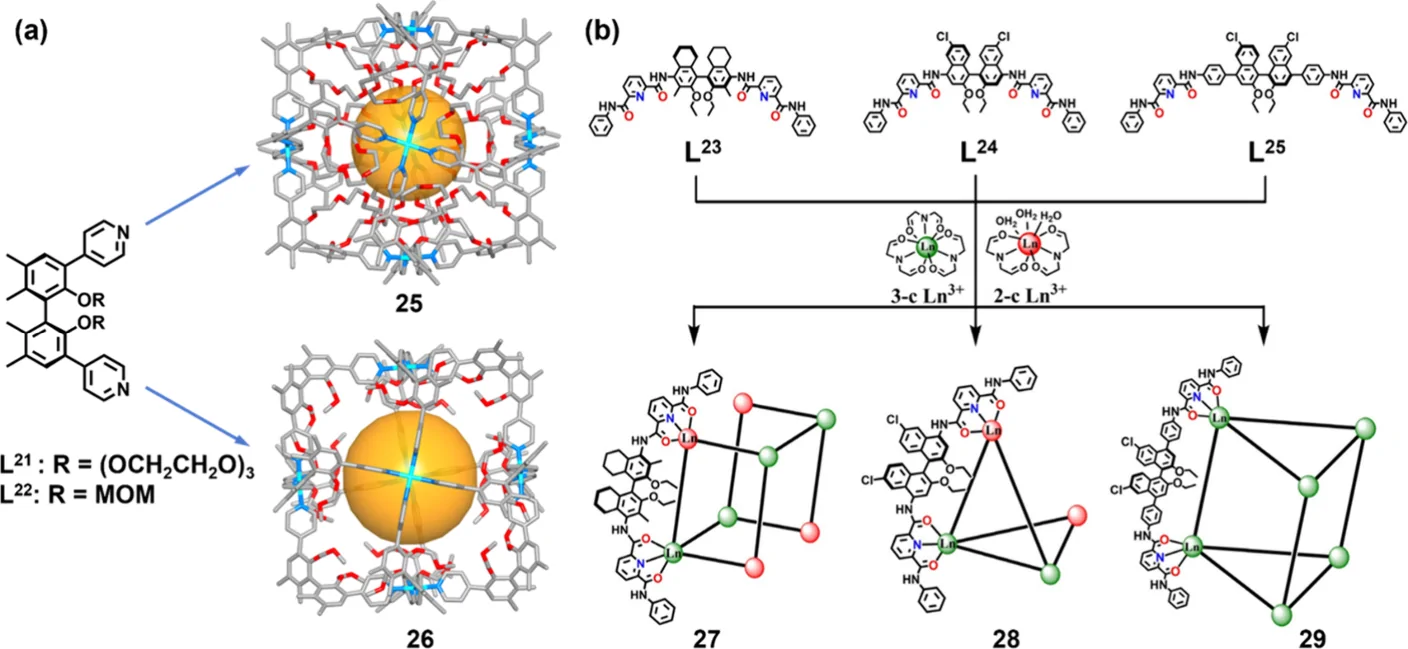
(a) Crystal structures of chiral cubic cages **25** and **26**. (b) Self-assembly of defective cages **27**–**29**. Reproduced with permission from ref 66. Copyright 2021, Wiley. Counterions and solvent molecules have been omitted for clarity; C: gray; N: blue; O: red; Pd: cyan.

##### 2.2.2.4 Other Chiral Cages

Based on our previous studies on tetrahedral and octahedral cages, we realized that traditional construction strategies often rely on highly symmetric rigid precursors, resulting in structures that are typically limited to classical Platonic or Archimedean polyhedra. To endow cages with more complex functions and adaptability, researchers have gradually turned their attention to unconventional configurations. The synthesis of intricate cages featuring multiple vertex types, missing edges, or nonclassical topologies (such as Johnson solids) by incorporating nonplanar ligands, exploiting coordination defects, or modulating structural isomerism has become a cutting-edge direction. Such structures not only break the conventional constraints of symmetry but also offer deformable frameworks and heterogeneous microenvironments, providing a new platform for precision host–guest chemistry and selective catalysis.

For example, we have reported the design and synthesis of defective chiral coordination cages using steric hindrance from organic linkers to control the coordination modes of metal centers. The assembly of Ln^III^ ions with sterically bulky enantiopure BINOL-derived ligands **L** ^**23**^ and **L** ^**24**^ afforded hexahedral and tetrahedral cages with two and one missing linkers of cages **27** and **28**, respectively (Figure 5b). In contrast, self-assembly of the less bulky ligand **L** ^**25**^ gave the regular but twisted triangular prism cage **29** without missing edges.^66^ Increasing the number of coordination sites in ligands further enhanced assemblies complexity. When the tetradentate TADDOL-based enantiopure ligand (**L** ^**26**^) was reacted with nickel­(II) salts in different organic solvents, three nonclassical concave cages of Ni_14_ **L** ^**26**^_8_ (**30**), Ni_18_ **L** ^**26**^_12_ (**31**), and Ni_24_ **L** ^**26**^_16_ (**32**) along with one regular convex cage Ni_6_ **L** ^**26**^_8_ (**33**) were obtained (Figure 6a). Notably, cages **31** and **32** possess suitable windows and cavities, enabling their use as chiral hosts for the adsorption and resolution of racemates.^67^

**Figure 6:**
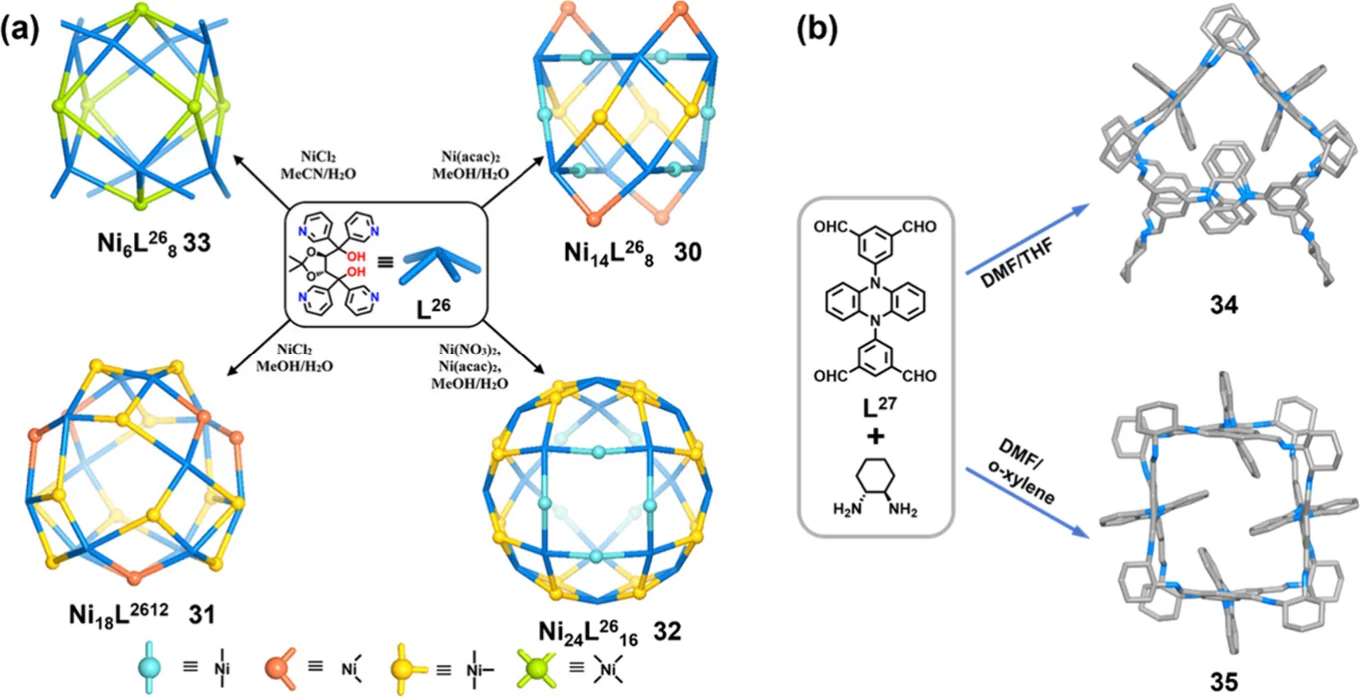
(a) Self-assembly of chiral TADDOL-based cages **30–33**. Reproduced with permission from ref 67. Copyright 2023 American Chemical Society. (b) Crystal structures of organic cages **34** and **35**. C: gray; N: blue.

To enhance the chemical stability of chiral cages, dynamic covalent bonds were introduced into the cage frameworks. Two complex homochiral porous [4 + 8] organic cages **34** and **35**, were synthesized using dihydrophenazine-derived ligand (**L** ^**27**^) and 1,2-cyclohexanediamine as building blocks (Figure 6b). Structural isomerism was observed, depending on the orientations of the four tetraaldehyde building blocks, resulting in an unprecedented Johnson-type *J*_26_ structure for **34** and a tetragonal prismatic structure for **35**. Furthermore, the imine linkages in these cages were reduced using NaBH_4_ to afford the amine isomers **34R** and **35R**, which hold great potential for asymmetric catalysis.^68^

In short, chiral cages have emerged as a versatile supramolecular platform that integrates well-defined three-dimensional cavities with tunable chiral microenvironments. As discussed in this section, significant progress has been made in the construction of chiral helicates, tetrahedral, octahedral, cubic, and nonclassical polyhedral cages through coordination-driven assembly and dynamic covalent chemistry. Strategies involving flexible or sterically bulky ligands, multinuclear metal clusters, and defect engineering have enabled the creation of cages with unconventional topologies and enhanced functionality. These diverse chiral cages not only enrich the structural library of supramolecular chemistry but also provide ideal confined spaces for enantioselective recognition, asymmetric catalysis, and chiral separation, as will be further elaborated in the following sections.

## 3 Chiral Application

Chiral cages and macrocycles hold great potential in synthetic chemistry and materials science, spanning applications from enantioselective recognition and asymmetric catalysis to smart chirality-induced switching and sensing. Although conventional chiral small molecules have made considerable progress in these areas, macrocycles and cages with confined cavities and tunable chiral environments offer unique microenvironments that enhance substrate recognition and reaction selectivity. In this section, we systematically present the applications of these chiral macrocycles and cages in three key directions, including chiral recognition, enantioselective separation, and asymmetric catalysis, demonstrating their potential to mimic or even surpass natural enzymes.

### 3.1 Chiral Recognition

Nature exhibits a remarkable specificity in the chiral recognition of biomolecules. In particular, chiral OH/NH functionalities have played an important role in the origin of homochirality in living systems, including DNA, RNA, peptides, and enzymes.^69–71^ In this context, we deliberately incorporated NH and OH functionalities into chiral supramolecular assemblies for the high discrimination of complex racemates. For example, metallacycle **8** and helicate cages **10** and **11**, which feature chiral NH functionalities, exhibit excellent recognition performance toward chiral isomers such as sugar acids, amino acids, and amines.^51,56,57^ Notably, when **8** was treated separately with l *-*valine, l *-*leucine, l *-*threonine, l *-*phenylalanine, and l *-*tryptophan (1.0 × 10^–3^ M), the fluorescence quenching rates for (*R*)-**8** were consistently faster than those for (*S*)-**8**. The calculated *K*_sv_(*R*-**8**)/*K*_sv_(*S*-**8**) values were 4.86 ± 0.37, 2.99 ± 0.23, 2.54 ± 0.19, 3.15 ± 0.24, and 2.46 ± 0.17, respectively.

Similar to chiral NH functionalities, chiral OH groups were also introduced into a chiral microenvironment to recognize chiral isomers. Helicate cages **12** and **14** were employed to discriminate the enantiomers of amino alcohols.^58^ The results showed that Benesi–Hildebrand plots for ΔΔ**12** in the presence of d- and l *-*leucinol in CH_3_CN followed a 1:1 binding model. The association constants *K*_a_ were found to be 4201 M^–1^ for d-leucinol and 1059 M^–1^ for l *-*leucinol, affording an enantioselectivity factor (EF) *K*_a_(*D*)/*K*_a_(*L*) of 3.97 ± 0.04 (Figure 7). Upon titration with d-leucinol, the quantum yield of ΔΔ**12** increased from 13.21% to 16.98%, while the fluorescence lifetime showed a slight change from 3.46 to 3.56 ns. These observations suggest that the enantioselective recognition likely proceeds via a static enhancement process, resulting from the formation of a helicate-amino alcohol adduct through a combination of hydrogen bonding and hydrophobic effects.

**Figure 7:**
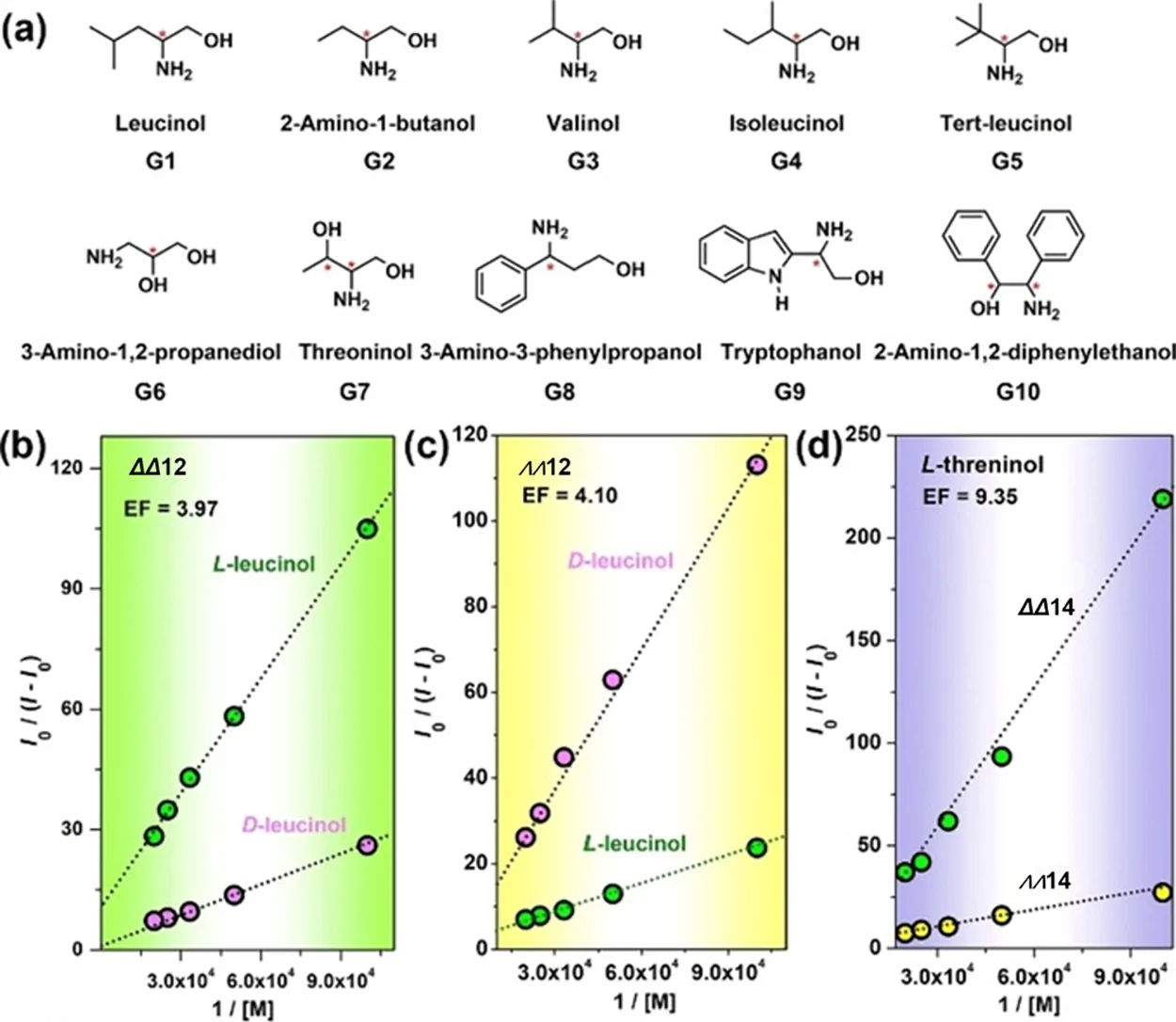
(a) Chemical structures of chiral amino alcohols used in this study. Benesi–Hildebrand plots of ΔΔ**12** (b) and *∧∧* **12** (c) titration with d- and l *-*leucinol. (d) Benesi–Hildebrand plots of ΔΔ**14** and *∧∧*14 titration with l *-*threninol. Reproduced with permission from ref 58. Copyright 2021, Wiley.

### 3.2 Chiral Separation

Whereas chiral recognition primarily centers on binding selectivity, chiral separation imposes more strict requirements by necessitating the physical isolation of a specific enantiomer from a mixture. The separation of enantiomers remains a persistent challenge due to their identical physicochemical properties. These chiral porous discrete assemblies may offer several key advantages for enantioselective separation: (1) their supramolecular frameworks can form well-defined crystalline nanochannels through highly directional noncovalent interactions; (2) the transmission of chirality from the nanoscale to the mesoscale is readily achieved within their supramolecular frameworks; (3) chiral discrete assemblies generally possess good solution processability. Guided by these structural advantages, we systematically evaluated the enantioseparation capabilities of these assemblies, progressing from fundamental single-crystal encapsulation studies to advanced continuous separation technologies.

Metallacycle **6** demonstrated excellent absorption capacity for (*R*)-2-butanol, achieving an enantiomeric excess (*ee*) value of 99.8%.^49^ The absolute (*R*)-configuration was unambiguously confirmed by single-crystal X-ray diffraction (Figure 8a). However, each host **6** was found to accommodate only one (*R*)-2-butanol molecule, indicating limited separation efficiency. To improve this, the larger cavity of metallacycle **7** was employed.^50^ **7** also yielded satisfactory enantioseparation results for (*R*)-2-butanol and (*R*)-3-methyl-2-butanol, with ee values of 98% and 95%, respectively. Moreover, the longer-chain 2-pentanol was also absorbed within the framework, affording 99% *ee* for the enantioselective encapsulation of the (*R*)-enantiomer. X-ray crystallography revealed that each **7** cavity encapsulated six (*R*)-2-pentanol molecules (Figure 8b). Similar enantioseparation behavior was observed for tetrahedral cage **16**, which preferentially bound the (*S*)-enantiomer of 2-butanol with 98% ee via cocrystallization.^59^

**Figure 8:**
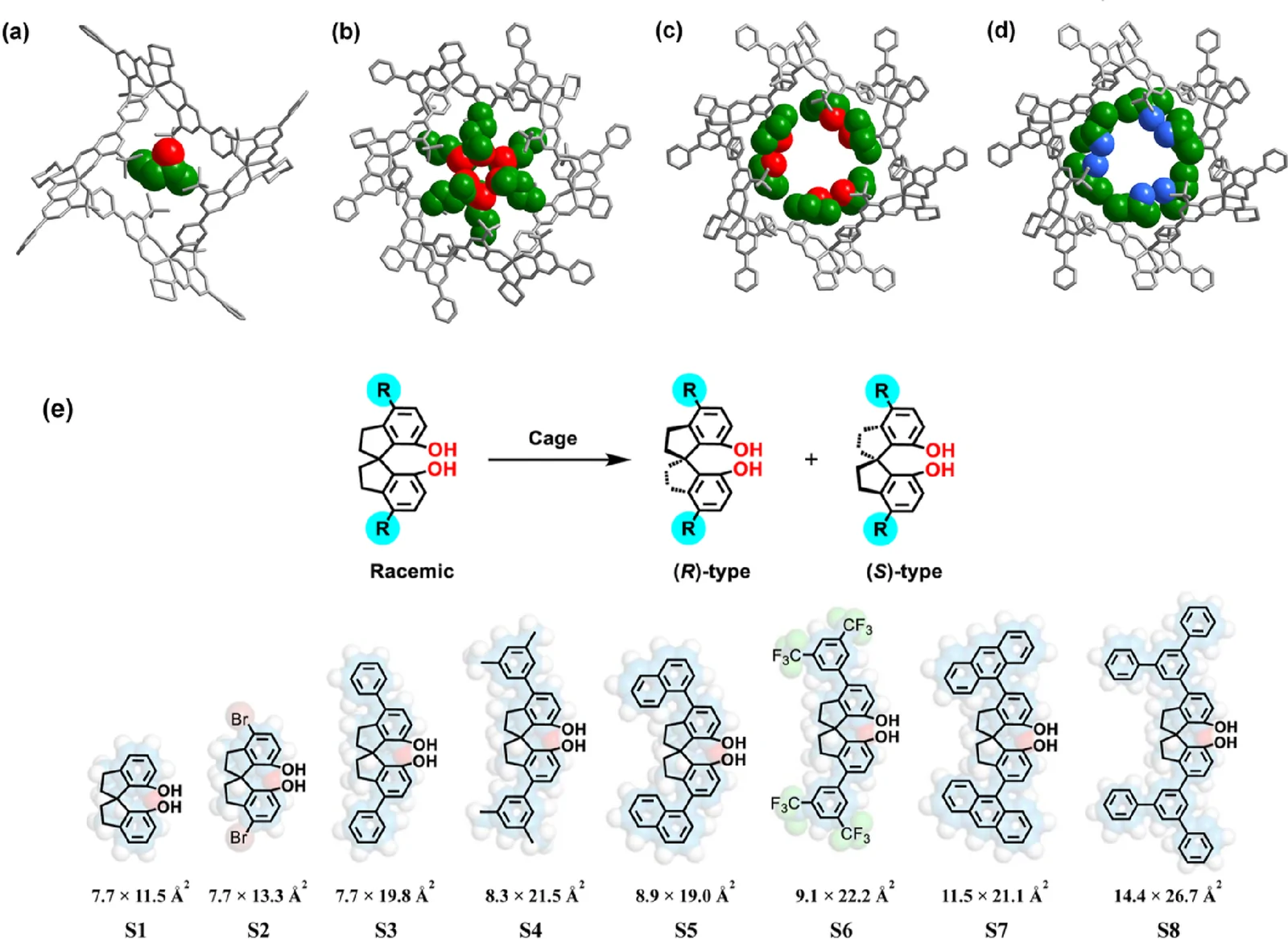
X-ray crystal structures of host–guest complexes: (a) (*R*)-2-butanol@**6**; (b) (*R*)-2-pentanol@**7**; (c) (*S*)-2-butanol@**8**; (d) (*S*)-2-butylamine@**8**. (e) Enantioselective adsorption and separation performances of the cages **30** and **31**. Reproduced with permission from ref 67. Copyright 2023, American Chemical Society.

Although simple alcohols serve as excellent models for probing effect of cavity size, the separation of pharmaceutically relevant chiral amines presents a greater challenge due to their flexible conformations. Leveraging the rigid chiral NH pockets of metallacycle **8**, we successfully resolved racemic 2-butylamine with an impressive ee of 99.4% (Figure 8c,d).^51^ Molecular simulations corroborated this experimental observation, revealing that the superior binding energy of (*S*)-2-butylamine with (*R*)-**8** (−74.41 kJ·mol^–1^ vs −71.71 kJ·mol^–1^) stems from optimal steric fit and stronger noncovalent interactions within the nanocavity.

In the field of efficient separation and purification of chiral enantiomers, chiral membrane separation exhibits unique potential due to its advantages of continuous operation and low energy consumption. While chiral solid-phase extraction plays an irreplaceable role with its high selectivity and excellent preconcentration and cleanup capabilities. They constitute key technological approaches for chiral recognition in complex systems. In this area, we have developed the chiral cages **18**–**20** capable of transmembrane transport of amino acids. By virtue of their high structural stability and excellent biocompatibility, the orientation-independent cages **18**–**20** can be embedded into lipid membranes, biomimetically functioning as single-molecule chiral channels for polar-residue amino acids. Specifically, hydroxyl-bearing cage **18** preferentially transports l *-*asparagine, while crown-ether-appended cage **20** spontaneously favors d-arginine transport. Furthermore, we utilized TADDOL-based cages **30** and **31** as chiral solid adsorbents for the solid-phase extraction separation of 1,1′-spirobiindane-7,7′-diol (spinol) derivatives. The results demonstrated that the (*D*)-**31**-based chiral stationary phase (CSP) was effective in the SPE separation of spinol derivatives, as exemplified by the resolution of **S4**, from which the (*S*)-enantiomer was obtained in 95% *ee*. The SC-XRD studies revealed that each (*L*)-**31** encapsulated three (*S*)-**S4** molecules from the racemic mixture, with the three guests arranged spirally around the *C*_3_ axis.

### 3.3 Asymmetric Catalysis

In the field of asymmetric catalysis, the confined chiral cavities provided by cages and macrocyclic hosts can effectively mimic enzyme catalytic properties, offering a unique platform to address the trade-off between activity and selectivity in asymmetric transformations. First, we attempted to settle the metal centers into the nanocavities to enhance the catalytic activity. For example, the MnIII­(salen)-based cage **23** exhibited good conversion (54–59%) and excellent enantioselectivity (91–99% *ee*) in the oxidative kinetic resolution (OKR) of racemic aromatic alcohols, and achieved 81–96% *ee* in alkene epoxidation with a catalyst loading of only 0.05 mol %.^63^ Its activity and selectivity were significantly superior to those of the MnIII­(salen) monomer, attributable to the confined cavity effect that suppresses intermolecular deactivation, stabilizes the catalyst, and increases reactant concentration. Inspired by this single-metal catalyst, we sought to mimic the multiactive-site architecture of natural enzymes by designing a heterometallic cage of **22MnCr**. By integrating Mn­(III) (for epoxidation) and Cr­(III) (for ring-opening) within a single nanocavity, this assembly catalyzes sequential asymmetric reactions with up to 99% ee (Figure 9a). In contrast, mixtures of the monometallic cages **22Mn** and **22Cr** or of the corresponding ligands exhibited lower conversion and *ee* values, with the differences becoming more pronounced at low loadings (0.005–0.05 mol %).^58^ This highlights the critical role of metal–metal synergy within the confined chiral microenvironment in enhancing catalytic performance.

**Figure 9:**
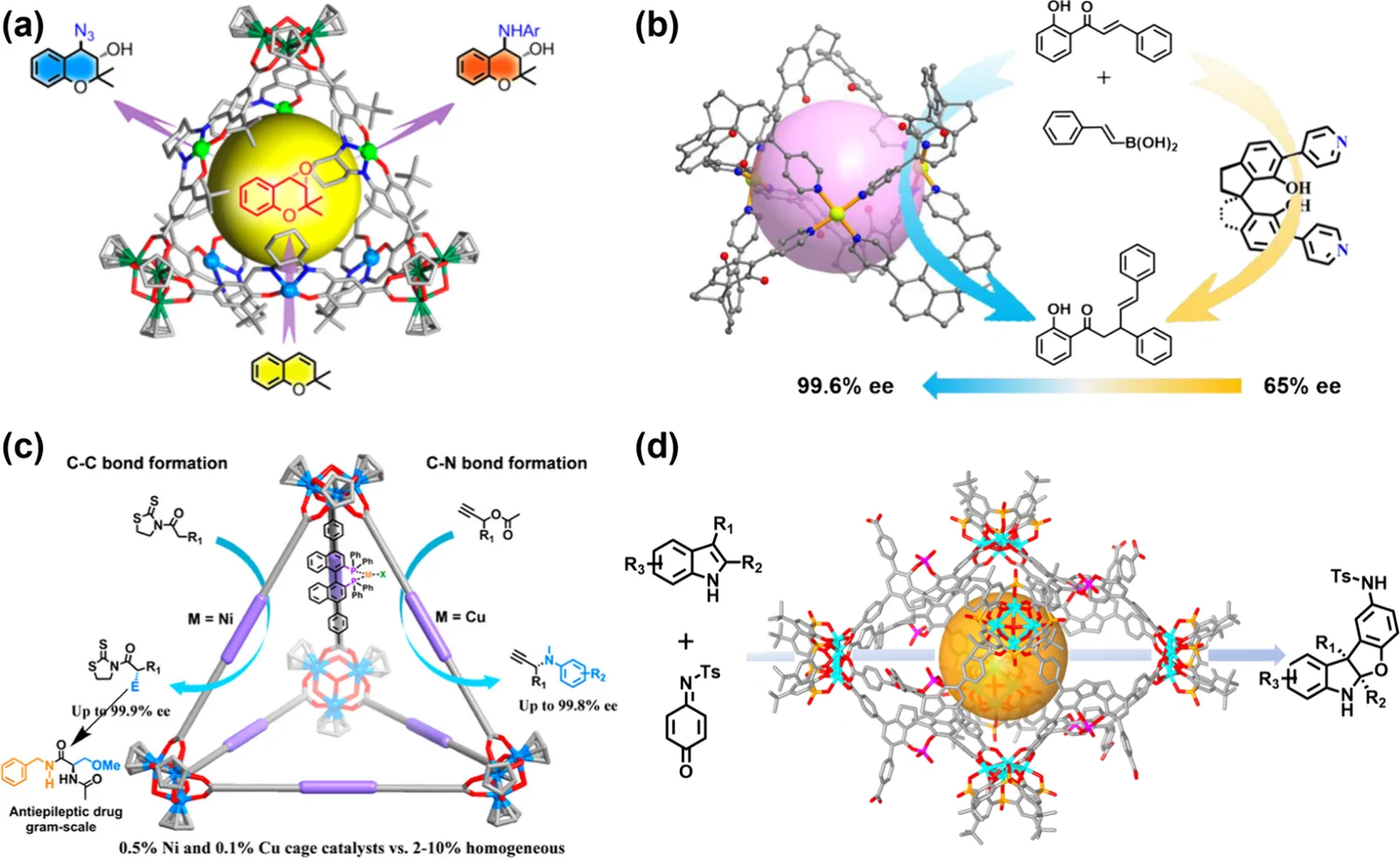
(a) Salen-based cages for sequential reaction. Reproduced with permission from ref 62. Copyright 2018, American Chemical Society. (b) SPINOL-based metallacycle for asymmetric conjugate addition reaction. (c) BINAP-derived cage for enantioselective C–C and C–N bond-forming reactions. Reproduced with permission from ref 61. Copyright 2026, American Chemical Society. (d) SPINOL-based cage for asymmetric [3 + 2] coupling reaction.

Furthermore, the postsynthetic metallization of OH or PPh_2_ moieties within chiral confined spaces endows chiral assemblies with asymmetric catalytic activity. For instance, upon treatment with Mg^2+^ or Ti^4+^ ions, metallacycle **2** becomes capable of enantioselectively catalyzing the conjugate addition of α,β-enones with styrylboronic acids, as well as the addition of diethylzinc to aromatic aldehydes, affording the desired products with 91–99.6% *ee* and 80–99.9% *ee*, respectively (Figure 9b).^47^ Similarly, introducing Ni­(II) or Cu­(I) into the BINAP-derived cage **21** yields metalated cage catalysts that promote highly efficient and enantioselective C–C and C–N bond-forming reactions, including alkylation and propargylic amination, featuring excellent functional group tolerance and achieving up to 99.9% *ee* (Figure 9c). The robustness and recyclability of these metalated cage catalysts further enable the scalable synthesis of pharmaceutically relevant compounds.^61^

As an alternative to catalytic metal sites, introducing Bro̷nsted acid sites into chiral cages offers a promising route to highly efficient supramolecular catalysts. In cage **24**, uncoordinated carboxylic and phosphoric acid groups point outward from the cavity and form intermolecular hydrogen bonds, leading to the assembly of porous hydrogen-bonded frameworks.^64^ The resulting crystalline solid contains two newly generated cage-type cavities, endowing it with high permanent porosity and structural robustness. Remarkably, this cage-based porous material, featuring regularly arrayed phosphoric acid catalytic sites, serves as an effective heterogeneous catalyst for the asymmetric [3 + 2] coupling of indoles with quinone monoamine, producing optically active benzofuroindoline derivatives in 91–99% *ee* (Figure 9d). Furthermore, it is capable of promoting asymmetric Friedel–Crafts alkylations of indole with aryl aldimines, enabling the atom-economical synthesis of enantiopure 3-indolylmethanamine derivatives in 89–99% yield and 90–99% *ee*. This work not only bridges our understanding of chirality transmission from the nano- to macroscale but also provides a robust strategy for preparing heterogeneous supramolecular catalysts.

## 4 Conclusions and Outlook

In this Account, we have summarized our endeavors over the past decade in establishing a rational design framework for discrete chiral supramolecular assemblies. Our journey began with simple chiral building blocks and evolved into the sophisticated construction of chiral metallacycles and cages. The core insight gathered from our studies is that the creation of confined chiral microenvironments is not merely a structural curiosity but a critical determinant of function, governing processes ranging from chirality transfer and amplification to enantioselective recognition and asymmetric catalysis. While coordination-driven self-assembly offers a facile route to structurally diverse chiral assemblies, the field is still in its infancy regarding the precise engineering of function. The translation of molecular chirality into global supramolecular chirality remains less well-established than in traditional organic molecules, presenting both a challenge and an opportunity.

Looking forward, we envision that the study of chiral discrete assemblies will continue to be a dynamic frontier in metallosupramolecular chemistry. Based on our experience, several critical developments are anticipated as below. First, advancing the rational design of chiral microenvironments remains a synthetic challenge. While we have achieved partial control through the “directional bonding” strategy, the rational design of building blocks that can precisely position functional groups within a confined cavity is still difficult. A key frontier lies in moving beyond classical Platonic polyhedra toward nonclassical, defective, and interlocked architectures as discussed in Section 2.2.2.4. These unconventional structures break the constraints of high symmetry and offer distinct advantages for future applications. Their intrinsic structural flexibility, multiple vertex types, and heterogeneous microenvironments can better mimic the dynamic and adaptive features of natural enzyme pockets, which often rely on subtle conformational changes and cooperative interactions to achieve high catalytic efficiency and selectivity. For enzyme-mimetic catalysis, the presence of missing linkers or unconventional topologies may generate accessible active sites, confined yet flexible nanospaces, and multiple recognition motifs, enabling substrate binding and transition-state stabilization that closely resemble natural enzymatic processes. Moreover, although inducing chirality using achiral building blocks is a promising avenue, its broader applicability remains restricted. Consequently, more intricate chemical engineering is required for this approach to achieve widespread adoption in this field.

Second, exploring the nano- to macro-scale transmission of chirality within hierarchically assembled structures is crucial. Hierarchical organization of discrete assemblies into extended architectures, such as two-dimensional layers and three-dimensional porous frameworks, enabling the translation of molecular chirality into collective properties for heterogeneous catalysis, chiral separation, and sensing. The emergence of exfoliable chiral 2D materials from discrete assemblies provides a particularly compelling pathway toward tunable chiral interfaces.

Third, compared to porous materials like MOFs or COFs, chiral discrete assemblies offer excellent solution processability and well-defined nanospaces, making them ideal candidates for biomimetic applications such as transmembrane transport and drug delivery. However, their practical deployment in biological systems is hindered by challenges in biocompatibility, water solubility, and structural robustness under physiological conditions. On the other hand, they are also expected to be useful in large scale enantioseparation. For example, we can integrate these dispersed materials into the membranes or thin films, enabling the enantioseparation of chiral drugs and fine chemical intermediates. By combining the solution processability of discrete assemblies with the continuous throughput of membrane technology, we aim to establish a sustainable and energy-efficient platform for industrial-level optical resolution.

Overall, chiral discrete supramolecular assemblies are evolving from structurally elegant architectures into a general platform for chiral functions. We hope that this Account not only showcases our specific achievements but also inspires a broader community of chemists to view supramolecular assemblies not merely as structural marvels, but as versatile platforms for developing adaptive, enzyme-mimetic materials with practical utility, thereby driving further advancements in this dynamic field.
